# Potential Use of Microbial Enzymes for the Conversion of Plastic Waste Into Value-Added Products: A Viable Solution

**DOI:** 10.3389/fmicb.2021.777727

**Published:** 2021-11-30

**Authors:** Muhammad Tamoor, Nadia A. Samak, Yunpu Jia, Muhammad Umar Mushtaq, Hassan Sher, Maryam Bibi, Jianmin Xing

**Affiliations:** ^1^CAS Key Laboratory of Green Process and Engineering, State Key Laboratory of Biochemical Engineering, Institute of Process Engineering, Chinese Academy of Sciences, Beijing, China; ^2^College of Chemical Engineering, University of Chinese Academy of Sciences, Beijing, China; ^3^Biofilm Centre, Aquatic Microbiology Department, Faculty of Chemistry, University Duisburg-Essen, Essen, Germany; ^4^Department of Chemical Engineering, Wah Engineering College, University of Wah, Wah Cantt, Pakistan; ^5^Chemistry and Chemical Engineering Guangdong Laboratory, Shantou, China

**Keywords:** PLA-PET waste, bioconversion, polyhydroxyalkanoates, circular economy, fuel oil

## Abstract

The widespread use of commercial polymers composed of a mixture of polylactic acid and polyethene terephthalate (PLA-PET) in bottles and other packaging materials has caused a massive environmental crisis. The valorization of these contaminants *via* cost-effective technologies is urgently needed to achieve a circular economy. The enzymatic hydrolysis of PLA-PET contaminants plays a vital role in environmentally friendly strategies for plastic waste recycling and degradation. In this review, the potential roles of microbial enzymes for solving this critical problem are highlighted. Various enzymes involved in PLA-PET recycling and bioconversion, such as PETase and MHETase produced by *Ideonella sakaiensis*; esterases produced by *Bacillus* and *Nocardia*; lipases produced by *Thermomyces lanuginosus*, *Candida antarctica*, *Triticum aestivum*, and *Burkholderia* spp.; and leaf-branch compost cutinases are critically discussed. Strategies for the utilization of PLA-PET’s carbon content as C1 building blocks were investigated for the production of new plastic monomers and different value-added products, such as cyclic acetals, 1,3-propanediol, and vanillin. The bioconversion of PET-PLA degradation monomers to polyhydroxyalkanoate biopolymers by *Pseudomonas* and *Halomonas* strains was addressed in detail. Different solutions to the production of biodegradable plastics from food waste, agricultural residues, and polyhydroxybutyrate (PHB)-accumulating bacteria were discussed. Fuel oil production *via* PLA-PET thermal pyrolysis and possible hybrid integration techniques for the incorporation of thermostable plastic degradation enzymes for the conversion into fuel oil is explained in detail.

## Introduction: Plastic Waste, an Enormous Ecological Emergency

Global plastic production, including additives and fillers, has increased dramatically since the 1950s. Between 1950 and 2019, 8,100 Mt. of plastic was produced worldwide. Plastic production grew from 2 Mt. in 1950 to 450 Mt. in 2019 (Shanmugam et al., 2020). Currently, only 4 Mt. of biobased or biodegradable plastics are produced globally. They are used in almost every industry and household for disposable items such as wraps, plates, cups, and spoons (Song et al., 2009). Synthetic plastics are traditionally made from refined petroleum products in a controlled environment. These heavy crude oil-based plastics have the potential to contribute to the loss of resources, climate change, and greenhouse gas emissions (Brandt, 2011). However, the microbial degradation of plastic is determined by chemical bonding, rather than the source monomers (Blanco et al., 2020). As common plastics are composed of multiple repeats of small monomer units, they have a high molecular weight. Biological resources can be used to obtain polymers, including polyethylene (PE), polypropylene (PP), polyvinyl chloride (PVC), and polyethene terephthalate (PET), which all start with similar monomers. These plastics are not biodegradable, despite including biobased components (Alshehrei, 2017). For example, plastic materials are made of high molecular weight polymers and additives. They are indispensable in many applications owing to their versatility; they are used in the packaging, construction, automotive, electronic, agriculture, home, leisure, and sports industries, among others (Zhang et al., 2018; PlasticsEurope, 2019).

The demand for readily available plastic items continues to grow as the world’s population grows and lifestyles change. Thus, synthetic plastic manufacturing dominates global industries. Globally, 350–400 million tons of plastic waste is generated each year (Roser, 2018), with this value predicted to be 3-fold higher by 2050 (Macarthur et al., 2016). As present, the largest global waste challenges are food waste and plastic waste (Su et al., 2020a; Nanda and Berruti, 2021). Contamination resulting from plastic use is a global problem that occurs in all land areas and oceans, with an estimated 580,000 pieces of plastic per square kilometer (Wilcox et al., 2015). A more recent United Nations-commissioned study discovered that over 600 species of organisms, ranging from bacteria to whales, have been damaged by marine plastic waste; most of those organisms became ill after ingesting the plastic (Bhuyan et al., 2021). The most common food packaging products are high-density polyethylene (HDPE) flexible plastic packaging, polystyrene (PS) styrofoam boxes, PP rigid plastic containers, and PET soft drink bottles (Lim et al., 2018). The difference between the plastic waste produced globally and recycled waste is presented in Table 1. Various solutions have been proposed to deal with plastic waste, including waste sorting, value-added recycling, energy recovery, product bans, and biodegradable plastic production (Su et al., 2020b). The separation of various types of plastic waste from food waste is impossible, and this is likely to persist for an extended time (Gala et al., 2020). Organic residues in plastic-contaminated food waste can contaminate the soil, water, and air with pathogens, greenhouse gases, and unpleasant aromas (Zhang et al., 2021). Plastic and food waste can act as a catalyst for the dispersion of disease-causing microbes, such as viruses (Zhang et al., 2020). The costs and time requirements associated with cultivating, maintaining, and extracting enzymes from microorganisms are somewhat high. The process of enzymatic microbial degradation usually comprises the breaking of polymer bonds to produce smaller monomer components that can be processed further (Koshti et al., 2018). Petroleum-based polymers degrade slowly and research into their biodegradation is in its infancy, despite recent studies examining the biodegradation of plastic waste using specialized microbial strains (Baskaran and Rajamanickam, 2019; Gunawan et al., 2020; Li et al., 2020). A critical research gap is the lack of well-characterized microbes capable of digesting plastic that can be employed as model organisms for further investigations. Consequently, scientists should focus their efforts on the discovery of plastic-degrading bacteria to provide further groundwork with respect to the rapid biodegradation of polymers (Sánchez, 2020; Carmen, 2021; Matjašič et al., 2021). The total plastic waste and recycling development over the last decade are presented in Table 1.

**Table 1:** Difference between total plastic waste and recycled waste.

Year	Total waste generation (Million tonnes)	Recycling (Million tonnes)	Difference (%)
2006	24.5	4.7	80.81
2007	24.6	5.0	79.67
2008	24.9	5.2	79.12
2009	24.1	5.5	77.18
2010	24.5	6.0	75.51
2011	24.8	6.3	74.60
2012	24.8	6.9	72.18
2014	25.8	7.7	70.16

The data shown in this table are summarized from “An analysis of European plastics production, demand and waste data” (PlasticsEurope, 2016).

According to “Plastics – the Facts (PlasticsEurope, 2016),” which examined data from 2006 to 2014, there has been a slight growth in waste generation and notable increase in recycling. However, the percentage difference between waste generation and recycling is still very large. This difference can be reduced by replacing plastic with biodegradable plastic and using biotechnology to enhance plastic biodegradation, which will reduce pollution and contribute to a healthy environment (PlasticsEurope, 2016). Only approximately 9% of all plastic is recycled after use, which is a major cause of global plastic pollution (Hopewell et al., 2009).

This review focuses on microbial hydrolase enzymes that have been shown to digest PET-polylactic acid (PLA) under anaerobic or aerobic conditions. PET and PLA are abundant and incredibly valuable materials that have a wide range of applications. Therefore, the development of technologies to valorize PET-PLA waste is needed to prevent plastic pollution and achieve a circular economy. Herein, we have discussed plastic biodegradation by *I. sakaiensis*, *Bacillus*, *Thermomyces lanuginosus*, *Candida antarctica*, *Triticum aestivum*, and *Burkholderia* spp., and *Leaf-branch compost cutinases* (LCC) for PET-PLA generated monomers that are directly upcycled into small molecules with added value, including cyclic acetals, 1,3-propanediol, and vanillin. *Pseudomonas stutzeri*, *Pseudomonas aeruginosa*, *Streptomyces setonii*, *Streptomyces badius*, *Rhodococcus ruber*, *Comamonas acidovorans*, *Butyrivibrio fibrisolvens*, and *Clostridium thermocellum* are the main bacterial species among the vast microbial populations linked to polymer degradation. Similarly, fungal species, including *Aspergillus flavus*, *Aspergillus niger*, *Fusarium lini*, *Mucor rouxii*, and *Pycnoporus cinnabarinus*, are discussed. We have also focused on fuel oil production using various methods, such as PLA-PET thermal pyrolysis/microwave-heated pyrolysis, self-made catalysts, fluidized-bed reactors, and in-house fabricated glass reactors. A possible hybrid integration technique to incorporate thermostable plastic degradation enzymes in the conversion into fuel oil will be explained in detail. This section discusses how biobased plastics can act as an alternative to fossil-based plastics. The challenges of PLA-PET waste bioconversion or thermal conversion into value-added products and fuel oils have been critically investigated.

## Microbial Enzymes: a Potential Solution for Green Recycling

Most high molecular weight synthetic polymers can be biodegraded by microorganisms. Microbes have the ability to break down highly crystalline polymers; however, their utilization in commercial plastics is restricted. Microbiomes commonly interact with abiotic elements, such as heat and light, to alter polymer structures and enzymatic attack (Lear et al., 2021). Plastics are biodegraded by bacteria, fungi, and algae found in landfill leachate, sewage sludge, and compost. These microorganisms can degrade both natural and synthetic polymers (Sankhla et al., 2020).

**Figure 1:**
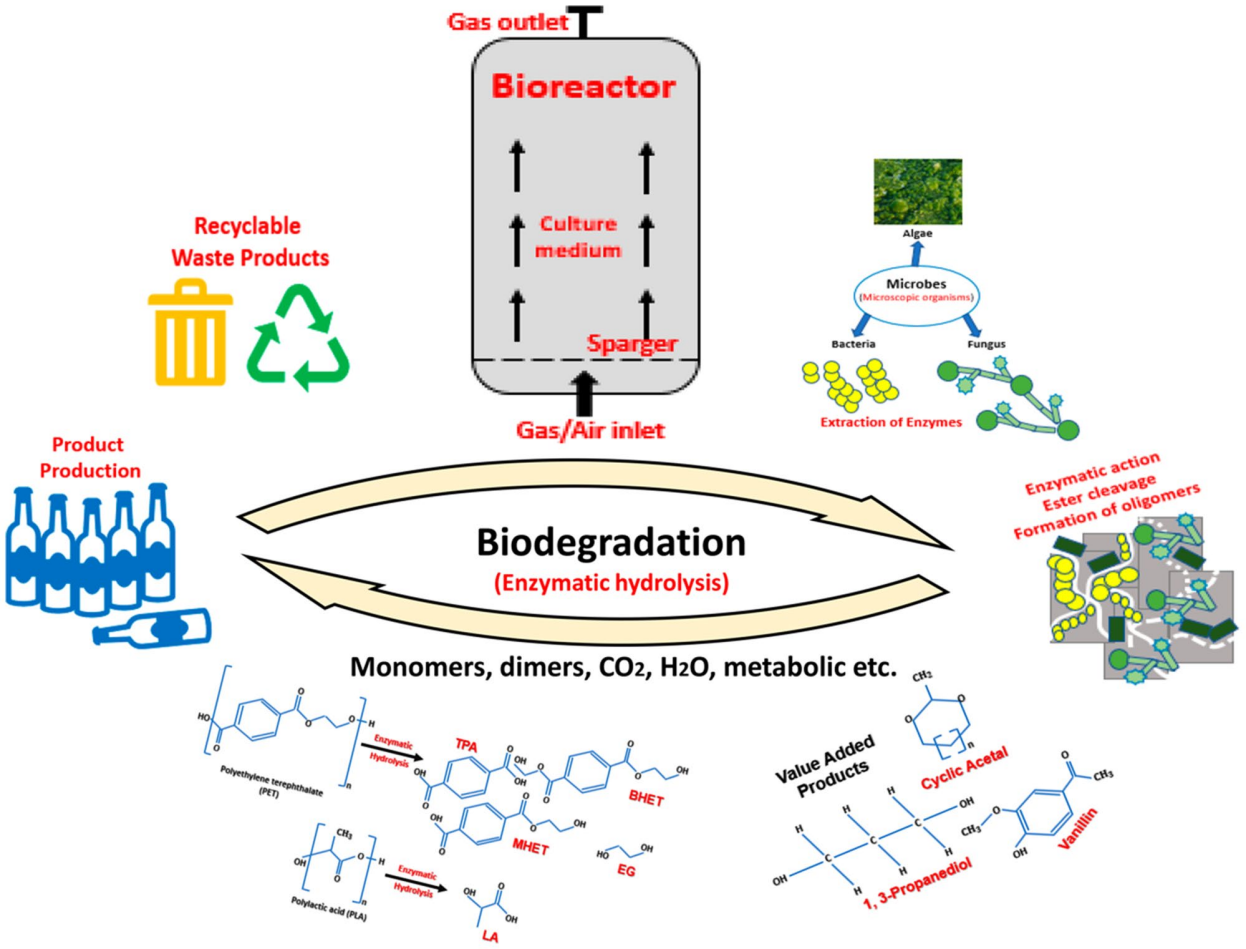
Schematic workflow of the biodegradation of plastic waste through enzymatic hydrolysis. The biodegradation of PLA-PET plastic waste is dependent on the diversity of the microorganisms and the environmental/experimental conditions. At a commercial level, this biological process occurs in bioreactors. The culture medium in the bioreactors results in the production of enzymes by the microbes. After excretion of the enzymes, they attach to the external surface of the plastic. As time passes, owing to their enzymatic activity, cracks appear on the polymer surface, subsequently causing it to decompose into lower molecular weight units, i.e., monomers and the other value-added products. The recycled monomers can be reused as a resin for polymer production.

Microbial biomass is a waste product of biodegradation. The waste products are the outcome of using organic polymers as energy and growth substrates, as shown in Figure 1. Enzymatic energy harvesting by microorganisms efficiently destroys natural polymers. Surprisingly, bacteria have evolved also these ancient decomposition pathways for use to degrade artificial plastic polymers. For example, *Ideonella sakaiensis 201-F6* was shown to grow solely on PET. *PETases* and *MHETases* are two new enzymes catalyzed in nontoxic monomers by PET hydrolysis [for example, terephthalic acid (TPA) and ethylene glycol (EG); Yoshida et al., 2016]. In addition to *I. sakaiensis*, which biologically produces *PETase*, several bacterial systems, including *Bacillus* and *Escherichia*, have been studied as overexpressing *PETase* enzymes (Huang et al., 2018). *Cutinase*, *lipase*, and *esterase* are the most common enzymes studied for the degradation of synthetic oligomers or polymers (Danso et al., 2018). Multiple enzymes and metabolic pathways are frequently involved in the breakdown of polymers (Taniguchi et al., 2019). Enzymatic hydrolysis in stirred tanks and membrane bioreactors are the most commonly used techniques (Pino et al., 2018). High solid loadings are seen as an alternative to improve the biodegradation processes, whereas bubble columns and gas-lift bioreactors are used for enzymatic hydrolysis (EH).

### Bacterial Degradation of PLA-PET Waste

Microbes degrade organic matter *via* enzymes. Carbon, nitrogen, and sulfur are transferred in elementary cycles *via* biodegradation and this procedure releases CO_2_, methane, and other biological components (Chandra and Rustgi, 1998). Microorganisms have been studied for their ability to decrease the strength of synthetic and natural polymers (Rujnić-Sokele and Pilipović, 2017). Biodegradability depends on the environment, because microorganisms and biodiversity are diverse. Plastic biodegradability is affected by the characteristics of both the microbe and the polymer (Tokiwa et al., 2009). The diversity of microbes that can degrade plastic is affected by various circumstances, including ambient conditions, temperature, and pH. In terms of environmental conditions, the moisture content of the soil is critical for microorganism growth. The soil’s water content is essential for microbial activation (Briški and Domanovac, 2017). The hydrolytic cleavage of microorganisms increases as the moisture content increases. Temperature and pH are essential elements in the breakdown process (Karigar and Rao, 2011; Mohanan et al., 2020). In addition to ambient conditions, temperature, and pH, microbial diversity also fluctuates. The soil moisture content is critical for microorganism growth (Jin and Kirk, 2018; Ratzke and Gore, 2018).

Under certain conditions, the chemical structure of degradable plastics will be changed significantly. In biodegradable plastics, this change is caused by the action of natural microorganisms (ASTM International, 2017). In principle, all organic compounds are biodegradable. However, to achieve this, different factors must be considered. The degradation of bioplastics *via* microorganisms and their degrading enzymes has emerged as a method of solving the unintended waste stream generated by large-scale plastic production. PET-PLA is an abundant and valuable material with a wide range of applications throughout society. Unfortunately, the global economy is not yet ready to fully embrace the benefits of recycling plastics because of the lack of cost effective equipment to measure the quality of post-consumer PET-PLA trash. PET and other materials may be recycled from PET by utilizing technologies such as monomer recycling, although recycling focus on repurposing the monomers to make additional PET.

*Thermobifida fusca* KW3, a gram-positive thermophilic actinomycete secretes a highly hydrophobic carboxylesterase (TfCa) that can hydrolyze PET (Oeser et al., 2010). To enhance TfCa enzyme expression and avoid the formation of inclusion bodies, its codon usage and induction conditions were optimized. The optimization conferred a 4,000-fold increase in TfCa activity. The TfCa protein concentration was found to be 41.6mg/L after culture in a pH-controlled bioreactor (Samak et al., 2020). The hydrophobic synthetic cyclic PET trimer was hydrolyzed by a carboxylesterase from *Thermobifida fusca* KW3, a thermophilic *actinomycetes* at 60°C and pH 6 (Billig et al., 2010). The values of V_max_ and K_m_ for the hydrolysis were 9.3mmol^−1^ min^−1^ mg^−1^ and 0.5mM, respectively. The esterase demonstrated a strong preference for short- and medium-chain fatty acyl esters of *p*-nitrophenol. The enzyme retained 37% of its activity after culture at 50°C and pH 8 for 96h. The process used a standard three-element catalytic system that includes the amino acids serine, glutamic acid, and histidine.

Acero et al. (2011) expressed cutinases from *Thermobifida cellulosilytica* DSM44535 (Thc_Cut1 and Thc_Cut2) and *Thermobifida fusca* DSM44342 (Thf42_Cut1) in *Escherichia coli* BL21-Gold(DE3) to hydrolyze PET. Two cutinases from *T. fusca* KW3 were compared with PHA depolymerase (ePhaZmcl) from *Pseudomonas fluorescens*. pNP-acetate and pNP-butyrate exhibited k_cat_ and K_m_ values in the ranges of 2.4–211.9s^−1^ and 127–200μM and 5.3 and 195.1s^−1^ and between 1,483 and 2,133μM, respectively. The PET-based products had a high conversion rate to MHET and TA. The water contact angle (WCA) could also change as a result of the change in the hydrophilicity.

*Bacillus subtilis p*-nitrobenzylesterase (BsEstB) was isolated and confirmed to hydrolyze PET (Ribitsch et al., 2011). The expressed BsEstB of 55.2kDa showed a specific activity of 77 and 108U/mg after reaction with *p*-nitrophenyl acetate (PNPA) and *p*-nitrophenyl butyrate (PNPB), respectively. BsEstB demonstrated strong enzyme activity at 40°C and pH 7.0 and good stability for several days at pH 7.0 and 37°C. The half-life was 3days at 40°C and 6h at 45°C. The principal conversion products of PET films with a lower WCA were the TA and MHET products, which decreased from 68.2°±1.7° to 62.6°±1.1°.

*Thermobifida alba* (Tha_Cut1) cutinase was overexpressed for PET hydrolysis (Ribitsch et al., 2012b). The Cut1 sample demonstrated a considerable degree of active PET surface hydrolysis, which improved hydrophilicity, as observed by a decrease in the WCA from 87.7° to 45°. The PNPB and PNPA values were 2.72 and 6.03s^−1^ for *K_m_* and 213 and 1933μM for the *k_cat_*, respectively. Tha_Cut1 primarily released 2-hydroxyethyl benzoate after interaction with bis(benzoyloxyethyl)terephthalate (3PET). Unlike the well-studied *Humicula insolens* cutinase (HiC), which preferably liberates 3PET terminal benzoic acid, this takes up all the carbon, oxygen, and nitrogen, rendering it very toxic to the environment.

In *E. coli*, the enzyme Thh_Est, which shares 87% homology with an esterase found in *T. alba*, was studied for its ability to surface-hydrolyze PLA and PET (Ribitsch et al., 2011). PET was digested by Thh_Est, which released TA and MHET in similar proportions (19.8 and 21.5mmol/mol of enzyme) without producing higher-order oligomers such as BHET. PLA was hydrolyzed, and lactic acid was released. Both the enzymatic and surface hydrolysis processes of PET and PLA caused an increase in hydrophilicity; the WCA decreased from 90.8° and 75.5° to 50.4°, respectively, and the water droplets spread entirely over the surface.

Tcur1278 and Tcur0390 from *Thermomonospora curvata* DSM43183 were found to have 61% sequence identity with the enzyme from *T. fusca* (Wei et al., 2014). The optimal reaction temperatures of Tcur1278 and Tcur0390 were 60 and 55°C, respectively. It was found that the optimum pH of both enzymes was 8.5. After incubation for 60min at 55°C, Tcur1278 retained more than 80% of its initial activity, and Tcur0390 had less than 10% of its initial activity. At reaction temperatures of up to 50°C, Tcur0390 was exhibited more effect hydrolysis of poly (ε-caprolactone) and PET nanoparticles than Tcur1278. At 55 and 60°C, only Tcur1278 exerted hydrolytic activity on PET nanoparticles. In molecular dynamics situations, Tcur1278 was demonstrated to have superior thermal stability, which is likely to be the main reason for its greater hydrolytic activity against PET.

The cutinase Cut190, from *Saccharomonospora viridis*, was expressed in *E. coli* (Kawai et al., 2014). The substitutions of Ser226 to Pro and Arg228 to Ser were shown to increase activity when operating at 65–75°C and pH 6.5–8.0 in the presence of 20% glycerol and Ca^2+^ ions. The mutant Cut190 could hydrolyze various aliphatic polyester materials. Yoshida et al. (2016) discovered a unique species that used PETase and MHETase to hydrolyze PET, enabling it to utilize plastic as its only energy and carbon source. The bacterium could therefore easily convert PET into TPA and EG, which was used for growth. PET film was hydrolyzed at 60°C using a dual enzyme system, including the KW3 strain of *Thermobifida fusca* TfCut2, and a metagenome-derived LC-cutinase (Barth et al., 2016). In the enzyme system, the amount of breakdown products formed was 2.4-fold greater when compared with TfCut2 after reaction for 24h.

Cutinase from *Humicola insolens* (De Castro et al., 2017) showed the best performance of 16 commercial lipases and was used for reactions with PET, solely or in combination with the efficient MHET-converting lipase from *Candida antarctica*. When two enzymes were used in combination, the concentration of the product was more than twice that when the enzyme was used alone, and the rate of terephthalic acid production was increased, reaching 9.36g/L. Carniel et al. (2017) screened 10 enzymes using BHET as the substrate and found that the combination of *Candida antarctica* lipase B (CALB) and HiC resulted in complete synergetic depolymerization of PET to TPA and a 7.7-fold increase in PET to TPA yield was achieved.

Four types of cutinase from *Thermobifida cellulosilytica* were employed to hydrolyze HMLS-PET cords (Vecchiato et al., 2017). -COOH of 0.51nmol mm^−2^ resulted in the highest degree of functionalization, with 1.35mM of soluble product was released after 72h. Compared with NaOH-based chemical hydrolysis, EH only solubilizes 0.7% of the polymer, without any reduction in mechanical characteristics or crystallinity. PET-TE membranes with a highly crystalline structure could be hydrolyzed by cutinases 1 and 2 from *T. cellulosilytica* and a fusion protein of cutinase 1 with the polymer-binding module from *Alcaligenes faecalis* (Thc Cut1 PBM) through a very narrow pore size distribution (Gamerith et al., 2017). By comparing the effects of two surface-functionalized versions of Thc_Cut1_PBM in the EH of PET-TE film on hydrophilic and hydrophobic surfaces, the influence of surface chemistry was also studied. The initial PET-TE membrane had the highest surface hydrolysis efficiency, resulting in a weight loss of 0.36%, which corresponded to the removal of approximately 3nm of PET from the entire porous membrane surface, which was well correlated with a 4-nm increase in the measured barrier aperture. A chemo-enzymatic treatment was developed to recover TPA and EG from PET (Quartinello et al., 2017). The chemical treatment, performed at 250°C and 40bar, converted PET into 85% TPA and other small oligomers. The hydrolysis in the second step using the HiC yielding 97% pure TPA, which was comparable with commercial synthetic grade TPA (98%). Biundo et al. (2018) improved the activity of an esterase (Cbotu_EstA) from *Clostridium botulinum* on PET by site-directed mutations in zinc-binding domain and deleted the domain consisting of 71 amino acids at the N-terminus. The zinc-binding domain of the native enzyme had 50-fold lower activity than the combination with truncations and substitution. Moreover, the kinetic parameters of Cbotu_EstA demonstrated a noticeable shift from water-soluble to insoluble polymer substrates.

The protein Sub1, a potential cutinase from *Streptomyces scabies*, was expressed in *E. coli*, purified, and characterized (Jabloune et al., 2020). Among the carbon short chain *p*-nitrophenyl esters, Sub1 had the strongest hydrolytic activity. The V_max_ was 2.36mol g^−1^ min^−1^ for *p*-NPB. The Sub1 esterase hydrolyzed PET, as shown by the release of TA. The addition of Triton to Sub1 on PET led to an increase in activity, and the Sub1 activity was found to be stable at 37°C for at least 20days. The expression of BhrPETase in *Bacillus subtilis* was 0.66g/L, and the biochemical characteristics of BhrPETase showed that BhrPETase had stronger hydrolytic activity on amorphous PET than LCC and *Is*PETase, as previously reported, as expressed under the same conditions (Xi et al., 2021). The purified BhrPETase has a melting temperature of up to 101°C, making it the most heat-resistant bacterial PETase ever characterized.

Polylactic acid depolymerase from *Paenibacillus amylolyticus* showed degradation activities against PLA (Akutsu-Shigeno et al., 2003). Lactic acid was found to be a degradation product of PLA. The gene encoded 201 amino acid residues, including the conserved pentapeptide Ala-His-Ser-Met-Gly, which is present in the lipases of mesophilic *Bacillus* species. Kawai et al. (2014) (Kawai, 2010) reported that microorganisms and PLA depolymerases are capable of decomposing PLA. The finding offsets the fact that PLA is highly vitrifiable and that microbes capable of decomposing PLA can do so at temperatures as low as 37°C. In this case, members of the *Nocardiaceae* family are the putrefying microorganisms here. The *Amycolatopsis* PLA depolymerases were isolated and cloned as serine proteases. The thermophilic lipases used to make the PLA were derived from *Bacillus thermophilus* strains that could grow at 60°C. Enzymes such as proteases and lipases are widely used in industrial settings as PLA depolymerases. Protease-type depolymerases were enantioselective for L-PLA, but lipase-type depolymerases were enantioselective for D-PLA. Consequently, two unique types of PLA depolymerases degrade the lipases and proteases. The EH conditions of PLA fibers were optimized with lipaselipases from different sources (Lee and Song, 2011). *Pseudozyma antarctica* JCM 10317 from the *Pseudozyma* genus (PaE) showed high breakdown potential for biodegradable polymers (Shinozaki et al., 2013). Poly (butylene succinate-co-adipate) activity against phthalate esters (PaE) was 54.8±6.3U/mg. Moreover, it was found that PaE could also degrade PBS, PBSA, and PLA solid films.

Tchigvintsev et al. (2015) performed a search for novel carboxylesterases inside three marine metagenomic gene banks. They found 23 active clones, five of which were extremely active esters and, at 5°C, exhibited an ability to target both α-naphthalene and *p*-nitrophenyl esters. MGS0010 exhibited a 2-fold increase in activity in the presence of 3.5M NaCl or KCl; however, the other four esterases were inhibited to various degrees. Esterases may also hydrolyze PLA polyester substrates, suggesting that they may be suitable for depolymerizing polyester. To enhance the amount of hydroxyl and carboxylic groups on the polymer chains, *H. insolens* cutinase (Pellis et al., 2015) was employed to hydrolyze PLA film. Another change in WCA was found, as it decreased from 74.6 to 33.1°, and roughness and waviness were 10 times higher than in than the control samples.

The structures of two native cutinases from *Thermobifida cellulosilytica*, named Thc_Cut1 and Thc_Cut2, as well as of two variants were investigated by Ribitsch et al. (2017). They showed different activities toward PLA. Although, the crystal structure analysis did not reveal significant conformational differences, unique SAXS scattering data collected from enzymes in the solution showed significant surface charge differences among the different cutinases. The differences in surface electrostatic potential and hydrophobicity could explain the different binding patterns of the four cutinases on PLA, and thus explain their different activities. Est119 from *Thermobifida alba* AHK119 can degrade polymer substrates, including PLA. The PLA-degrading mechanism by Est119 was clarified by Kitadokoro et al. (2019), who found that the amino acid residues responsible for substrate interaction were substantially conserved in the enzymes of *Ideonella sakaiensis*, including cutinases and PETase. The bacterial enzymes in literature for the biodegradation are briefly mentioned in Table 2.

**Table 2:** Summary of polylactic acid and polyethene terephthalate (PLA-PET) degrading enzymes, their microbial sources, and the value-added products released from the biodegradation process.

Microorganism	Enzyme	Plastic waste substrate	Value-added product release	Reaction conditions	References
*Thermobifida fusca* KW3	TfCa	PET nanoparticle	TPA	50°C	Oeser et al., 2010
*Thermobifida alba*	Tha_Cut1	3PET	2-hydroxyethyl benzoate	50°C	Ribitsch et al., 2012b
*Ideonella sakaiensis*	PETase	PET	MHET, TPA, and BHET	30°C	De Castro et al., 2017
*Thermobifida fusca* KW3	Carboxylesterase	Cyclic poly(ethylene terephthalate) trimers	MHET, TPA, and BHET	50°C	Billig et al., 2010
*Thermobifida cellulosilytica* DSM44535	Thc_Cut1, Thc_Cut2	3PET PET films	MHET, TPA	50°C	Acero et al., 2011
*Thermobifida fusca* DSM44342	Thf42_Cut1	3PET, PET films	MHET, TPA	50°C	Acero et al., 2011
*Thermobifida fusca* KW3	TfCa, TfCut2, LC-cutinase	PET films	MHET, TPA	60°C	Carniel et al., 2017
*Candida antarctica* *Humicola insolens*	16 commercial lipases and cutinases	Amorphous PET bottle	TPA, MHET, and BHET	60°C	Vecchiato et al., 2017
*Candida antarctica* *Humicola insolens*	CALB and (HiC)	Non-carbonated mineral water bottles	TPA, MHET, and BHET	37°C 50°C 60°C	Gamerith et al., 2017
*Thermobifida cellulosilytica*	Thc_Cut1, Thc_Cut2 Thc_Cut2_DM (double mutant Arg29Asn_Ala30Val of Thc_Cut2), Thc_Cut2_TM (triple mutant Arg19Ser_Arg29Asn_Ala30Val of Thc_Cut2)	PET fabrics and PET 1440/2 GXD cords	TPA, MHET, and BHET	50 60 70°C	Quartinello et al., 2017
Bacterium HR29	BhrPETase	PET powder	TPA, MHET, and BHET	30–80°C	Xi et al., 2021
*Bacillus subtilis*	*p*-Nitrobenzylesterase	3PET	TPA benzoic acid 2-hydroxyethyl benzoate, MHET	40°C	Ribitsch et al., 2011
*Thermobifida halotolerans*	Thh_Est	PET and PLA films	TPA, MHET	50°C for PET, 37°C for PLA	Ribitsch et al., 2012a
*Thermomonospora curvata* DSM43183	Tcur1278 and Tcur0390	PET nanoparticles		50°C	Wei et al., 2014
*Fusarium oxysporum*	Cutinase FoCut5a	Commercial PET woven fabric with tricot knit	TPA, MHET, and BHET	40°C	Dimarogona et al., 2015
*Fusarium oxysporum*	FoCut5a	PET fibers	TPA	40°C	Kanelli et al., 2015
*Thermobifida cellulosilytica*	Thc_Cut1_PBM	PET track-etched membranes	TPA, MHET, and BHET	50°C	Gamerith et al., 2017
*Humicola insolens*	HiC	Raw PET fiber	TPA	50°C	Quartinello et al., 2017
*Streptomyces scabies* EF-35 (HER1481)	Sub1	PET ground granules	TPA	37°C	Jabloune et al., 2020
*Aspergillus niger*	LAN	PLA fiber	Lactic acid	40°C	Lee and Song, 2011; Nakajima-Kambe et al., 2012
*Candida cylindracea*	LCC	PLA fiber	Lactic acid	40°C	Lee and Song, 2011
*Candida rugosa*	LCR	PLA fiber	Lactic acid	45°C	Lee and Song, 2011
*Humicola insolens*	HiC	PLA film	Lactic acid	37°C	Pellis et al., 2015
*Alcanivorax borkumensis*	ABO2449	PLA10 PLA18	Lactic acid monomers, dimers, and larger oligomers	30°C	Hajighasemi et al., 2016
*Rhodopseudomonas palustris*	RPA1511				
Rhizosphere bacteria and fungi		PLA and PET film		12.5°C	Janczak et al., 2020
*Pseudozyma antarctica* JCM10317	PaCLE1	PLA film	Lactic acid	30°C	Shinozaki et al., 2013
*Clostridium botulinum*	Cbotu_EstA	PET film	TPA, MHET	50°C	Biundo et al., 2018
*Paenibacillus amylolyticus* TB-13	PlaA	PLA samples with weight-average molecular weights	Lactic acid	37°C	Akutsu-Shigeno et al., 2003

3PET, bis(benzoyloxyethyl) terephthalate.

### Fungal Degradation of PLA-PET Waste

The fungal kingdom comprises an exceedingly diverse and fascinating eukaryotic collection of aerobic microorganisms varying from simple yeast cells to highly mycelial molds (Rossman, 1995). Single-celled or pseudomycelia growth is the standard for yeasts, although molds usually grow as mycelia. Because fungi, like bacteria, decompose and mix with organic substances, they are vital to the breakdown of the C–C bonds in the biosphere. Different species of bacteria and fungi can indeed grow in conditions with less moisture and in solutions with a lower pH, which helps organic materials decompose (Spellman, 2007). Fungi are incredibly active in decomposing natural polymeric materials owing to their extracellular multienzyme complexes. They are also capable of rapidly colonizing and penetrating substrates *via* their hyphal systems and transporting and redistributing nutrients within their mycelium *via* their hyphal systems (Matavulj and Molitoris, 2009). A diverse range of fungal strains from various classes, ecologies, and morphologies has been demonstrated to destroy plastics.

Fungi are highly capable of degrading many materials, including lignin, and are used in remediating contaminants (Fritsche and Hofrichter, 2005), as indicated in Table 3. New studies have indicated that the *Aspergillus genus* is the most prevalent of all the fungal groups appropriate for the biodegradation of synthetic plastics. *Aspergillus clavatus*, *Aspergillus fumigatus*, and *Aspergillus niger* are three different species of *Aspergillus* that have been found in terrestrial habitats around the world and are known to degrade PE, PU, and PP (Abraham et al., 2016). *Neosartorya*, *Amorphoteca*, and *Talaromyces* were deserted from soil polluted with petroleum and demonstrated to be promising hydrocarbon degradation organisms (Chaillan et al., 2004). In addition, it was discovered that the collectively, a number of individuals of fungi, namely *Penicillium*, *Cephalosporium*, and *Aspergillus*, are capable degraders of crude oil hydrocarbons (Singh, 2006). *Alternaria solani*, *Fusarium solani*, *Spicaria* spp., *Geomyces pannorum*, *Phoma* sp., and *Penicillium* spp. are additional fungal species with a high degree of plastic degradability (Sowmya et al., 2015). Contrary to popular belief, different fungal consortia have been proven to destroy various polymers, such as PET and PLA, through synergistic action (Ghosh et al., 2019; Nikolaivits et al., 2021). Before eventual depolymerization, as well as their potential to release hydrophobin for improved hyphal adhesion to hydrophobic substrates, fungal hyphae are critical in both the initial colonization and the subsequent depolymerization of the fungus (Wang et al., 2018; Quarantin et al., 2019). Pretreatment factors such as exposure to light and temperature and acid pretreatment and various additives have been used to enhance fungal biodegradation of plastics (Montazer et al., 2020). The hydrolysis of PLA fibers by lipases was greatest at 40°C for 60min at pH 7.5 for *A. niger* lipase, 40°C for 120min at pH 8.0 for *Candida cylindracea* lipase, and 45°C for 120min at pH 8.0 for *Candida rugosa* lipase. Lipase from *Aspergillus niger* MTCC 2594 was 53.8-fold purified by hydrophobic chromatography after interaction with octyl sepharose, and the enzyme revealed two protein bands with apparent molecular masses of 35 and 37kDa (Nakajima-Kambe et al., 2012). The lipase had maximum activity at pH 7.0 and 37°C, and was stable between pH 4.0 and 10.0, as well as temperatures up to 50°C. Olive oil, as a substrate, yielded values of K_m_ and V_max_ of 3.83mM and 32.21μmol/min/mg, respectively. The lipase has the potential to degrade PLA and poly(ε-caprolactone). A *Fusarium oxysporum* cutinase, FoCut5a, was expressed in *E. coli* BL21 (Dimarogona et al., 2015). The highest production of recombinant FoCut5a was achieved using periplasmic expression at 16°C. The X-ray structure was determined at 1.9Å resolution. The crystal structure was highly similar to that of *Fusarium solani* cutinase. FoCut5a showed absolute activity at 40°C and pH 8.0, while it was active on three p-nitrophenyl synthetic esters of aliphatic acids, with the maximum catalytic efficiency for the hydrolysis of butyl ester. FoCut5a cutinase is capable of modifying the surface of PET fabrics. The optimal parameters were found to be 40°C, pH 8, and 1.92mg of enzyme loading per gram of fabric (Kanelli et al., 2015). The analysis of tensile test and dyeability proved that the surface treatment did not harm the quality of the original material. The S-2 strain of *Cryptococcus* sp. had remote similarity to cutinases, but not lipases (Masaki et al., 2005). This enzyme can degrade PLA and other biodegradable plastics such as poly(butylene succinate) and poly(3-hydroxybutyrate).

**Table 3:** Plastic biodegradation using different enzymes derived from fungi.

Microorganism	Enzyme	Plastic waste substrate	Value-added product	Reaction conditions	References
*Tritirachiium album ATCC 22563*	Moniliaceae	PLA film	ND	Liquid culture, 30°C	Jarerat and Tokiwa, 2001
*FsC*	*Fusarium solani pisi*	lcPET (7%) and bo-PET (35%)	ND	30–60°C	Griswold et al., 2003; Yoshida et al., 2016a
*Humicola insolens (HiC)*	Thermomyces	lcPET (7%) bo-PET (35%)	ND	30–85°C	Ribitsch et al., 2012b
*Fusarium oxysporum strain (LCH 1)*	Nectriaceae	PET fibers	TA		Nimchua et al., 2007
*F. solani f*. sp. *pisi DSM 62420*	Nectriaceae	PET fibers	TA		Nimchua et al., 2007
*Tritirachium album ATCC 22563*	Moniliaceae	PLA film	ND	30°C	Jarerat and Tokiwa, 2001
*Rhizopus delemar*	Mucoraceae	PET copolymers with dicarboxylic acids	ND	37°C	Nagata et al., 1997
*P. roqueforti*	Trichocomaceae	PLA	ND	Liquid cultures	Torres et al., 1996
*Clonostachys rosea* and *Trichoderma* sp.	Bionectriaceae and Hypocreaceae	PLA	TA	Low-temperature operation, liquid culture, and laboratory environment	Urbanek et al., 2017
*Penicillium citrinum*	Ascomycete	PET pellets	BHET, MHET, TPA		Liebminger et al., 2009
*Candida cylindracea*	Lipases	PET nanoparticle	ND		Ma et al., 2011
*Saccharomonospora viridis* AHK190	cutinase Cut190 S226P/R228S	Amorphous PET film	TPA, MHET, BHET	65–75°C	Kawai et al., 2014
*Cryptococcus* sp. strain S-2.	CLE	High-molecular-weight compound polylactic acid	Lactic acid	30°C	Masaki et al., 2005

ND, not detected.

### Algae as a Promising Candidate for Plastic Degradation

Microalgae are unicellular photosynthetic organisms capable of converting water and CO_2_ into nutrients such as proteins, amino acids, lipids, polysaccharides, and carotenoids. They are a good candidate for a “microbial factory” because they can use readily accessible materials, such as inorganic nutrients (CO_2_), water, sunlight, and wastewater, with little or no effort. The identification of algae and their toxins that can biologically degrade polymeric materials can help reduce white pollution (Bhuyar et al., 2019). In contrast, bioplastics made from algae offer the same qualities as petroleum-based polymers while being naturally biodegradable (Chia et al., 2020). They can be grown on ponds and do not require specialized fertile land or freshwaters. Thus, microalgal biomass is an ideal low-cost bioprocess system for the production of PHA (Arora et al., 2021). Among them, only a few eukaryotic algae, such as *Chlorella*, have been reported to produce PHA (Roja et al., 2019). Cyanobacteria are some of the oldest and first photoautotrophic microorganisms on earth. They contribute to the global carbon/oxygen cycle and helped to evolve life by creating an oxygen-rich environment. The phycocyanin pigment confers their characteristic blue-green color, hence the name blue-green algae (Kaewbai-Ngam et al., 2016). The growth of microalgae in a closed laboratory photobioreactor, wastewater, or open ponds can yield higher biomass. This biomass can be harvested and processed for use as a substrate by other heterotrophic bacteria and produce PHA (Rahman et al., 2014). Microalgae cultures can use inorganic nitrogen and phosphorus for growth, making them a perfect solution for tertiary and quaternary treatments. Moreover, their ability to remove heavy metals and some toxic organic compounds prevents the secondary pollution (Abdel-Raouf et al., 2012). Microalgae, like bacteria, are a biological pollutant owing to their endotoxins and carbon demand (Yan et al., 2016). Microalgae grow in natural and laboratory environments with bacteria in mutually beneficial relationships. Bacteria benefit from the oxygen and extracellular substances of microalgae and return the favor with CO_2_ and vitamins (Han et al., 2016). Organic pollution can harm water quality in many ways. Algal wastewater treatment systems are now widely accepted as an effective conventional treatment system. Algal wastewater treatment systems are a significant low-cost alternative to complex, expensive treatment systems, particularly for municipal wastewater purification (Sen et al., 2013). *Phaeodactylum tricornutum*, a photosynthetic chassis, produces a microbial cell factory that develops an engineered form of *PETase*, which is secreted into the surrounding culture medium to degrade PET in aquatic environments (Moog et al., 2019). To better predict how climate change will affect aquatic microbial ecosystems, we need to understand community resilience. Interlevel interactions in a mixed community of microalgae and bacteria have a large impact on system resilience (Sörenson et al., 2021). The potential of the microalgal species *Chlorella vulgaris* with pretreatment, marked by its effectiveness in cracking and changing polymers, aids the growth of microorganisms on fractured surfaces (Falah et al., 2020). They found that the microbial species could produce the biodegradation products found in PET, such as alkane esters, fatty acids, benzoic acid, and aromatics, and the most toxic biodegradation product is bis(2-ethyl hexyl phthalate). In total, 10 algal species belonging to nine families, seven orders, and 10 genera were identified from various polyethylene-degrading locations (Sharma et al., 2014). Microalgae produce enzymes that reduce reaction activation energy and weaken polymer chemical bonds, promoting biodegradation. Microalgae have the ability to convert plastics into metabolites such as CO_2_, H_2_O, and new cell biomass (i.e., mineralization).

Biomass gasification converts biomass into CH_4_, H, CO_2_, and NH_3_. The gas produced from this process can be used as fuel gas, engine fuel, or as a feedstock for other industrial processes (synthetic gas; Mckendry, 2002). Microalgae are productive (12.5kg m^−2^ year^−1^) compared with most other plants (Shay, 1993). As reported by Goudriaan et al. (2000), a 75% thermal efficiency was achieved in a 10kg dry weight h^−1^ pilot plant during the hydrothermal upgrading process of biomass. Bio-crude oil comprises approximately 45% of the whole feedstock weight and has a lower heat capacity of 30–35MJ kg^−1^, enabling it to work with diesel fuel and be improved further.

The liquefaction of biomass by direct heating is very well studied. A handful of methods rely on algal biomass for their technology. Minowa et al. (1995) performed direct hydrothermal liquefaction at temperatures of 300°C and pressures of 10MPa to achieve a moisture content of 78.4wt% and an oil yield of roughly 37% for *Dunaliella tertiolecta*. After 60min of holding time at 350°C, the oil obtained had viscosity in the range of 150–330mPa and calorific values in the range of 36–40kJ g^−1^. These values were close to those of fuel oil. As shown by the energy balance, the liquefaction method is a total energy producer.

Similarly, Demirbas (2010) achieved a maximum output of 64% dry wt. based on oil liquefaction at 300°C catalyzed by sodium carbonate in a study on oil recovery using *Botryococcus braunii*. The development of microalgae occurs much faster than on land. It is predicted that 20,000–80,000L of oil are produced annually per acre of algae, which is 7–31 times greater than the next best crop of palm oil. Open ponds, photobioreactors, and closed systems are industrial reactors for algae culture.

Aresta et al. (2005) examined various conversion strategies to manufacture microalgal biodiesel, including organic solvent extraction, supercritical CO_2_, pyrolysis, and hydrothermal technology. For the mining of microalgae biodiesel, the hydrothermal-liquefaction approach proved more successful than utilizing supercritical CO_2_. It is reasonable to assume that hydrothermal liquefaction is the most effective technological solution for producing biodiesel from algae among the selected methods.

The incorporation of nanosized materials, such as nanofibers, nanoparticles, nanotubes, nanosheets, and other nanostructures. A novel method incorporating aluminum sulfate-coated magnetic particles was highly effective at isolating microalgae species of the *Anabaena* and the *Aphanizomenon* genus from a mixed culture. The use of silver nanoparticles to increase the harvest of *Cyanothece 51,142* and *Chlamydomonas reinhardtii* microalgae resulted in a 3-fold increase (Pattarkine and Pattarkine, 2012; Safarik et al., 2016).

The following enzymes, used in conjunction with polymer nanoparticles such as polyacrylonitrile nanofibers, iron oxide, and nanoporous gold; silica nanoparticles with lipase from *Rhizopus miehei*; ferric silica and magnetic nanoparticles with lipase from *Burkholderia* sp.; and polyacrylonitrile nanofibers, successfully transformed plant oils into biodiesel *via* the transesterification process: *Pseudomonas cepacia* lipase, *Rizopus miehei* lipase, ferric silica, and magnetic nanoparticles with lipase from *Burkholderia* sp., and polyacrylonitrile nanofibers (Palaniappan, 2017). In addition, a nanomagnetic biocatalyst, composed of lithium-doped CaO, KF/CaO–Fe_3_O_4_, Fe_2_O_3_–CaO, and sulfate, integrated sodium titanate, zirconium, and carbon-based nanotubes and nanoparticles to yield up to 95% or more biodiesel from a variety of biomass and biodegradable waste (Liu et al., 2018).

Apart from increasing the efficiency of biodiesel production, a type of NP called zeolite has been employed commercially as an absorbent within the transesterification process. Zeolites consumed the unwanted moisture content (4–6%) from biodiesel. Mesoporous nanoparticles have also demonstrated a critical aptitude for regular microalgal-biofuel synthesis without cell lysis. Additionally, zeolites extract lipids from the microalgae cell membrane (Liu et al., 2008).

Moog et al. (2019) created a microbial cell factory utilizing the photosynthetic microalga *Phaeodactylum tricornutum* to produce and secrete an engineered version of *PETase* into the surrounding culture medium. Initial studies utilizing 3D culture weightless at 30°C examined *PETase* activity vs. PET and the copolymer polyethylene terephthalate glycol (PETG); the low-crystallinity PETG had an 80-fold higher turnover than bottle-grade PET. The breakdown products of the PET substrate were TPA and MHET.

*Chlorella pyrenoidosa* was grown to produce biofuel and bioplastic. The maximal growth took 10days. The strain grew faster in the presence of CO_2_. The chemically processed algal biomass produced bioplastic. The 14-day-old algal culture contained 27% polyhydroxybutyrate (PHB). This ensures that the microalgae-derived bioplastic is biodegradable. The microalgae detoxified chromium and nickel by 11.24 and 33.89% (Das et al., 2018). Table 4 summarizes the literature on biodegradable algae enzymes.

**Table 4:** Plastic biodegradation using different enzymes derived from algae.

Microorganism	Enzyme	Plastic waste substrate	Value-added product	Reaction conditions	References
*Tetraselmis chuii*, *Cylindrotheca fusiformis*, and *Nannochloropsis gaditana*	Xenic microalga	Extra organic carbon containing medium	Cell density of microalga found higher	Co-culture with strains of *Muricauda* sp., 33days	Han et al., 2016
*Phaeodactylum tricornutum*	Photosynthetic microalga	PET and the copolymer polyethylene terephthalate glycol (PETG)	TPA, MHET	PETase, cultured medium, 30°C	Moog et al., 2019
*Chlorella vulgaris*	Chlorophyta microalga	PET film	Alkanes, ester, fatty acids, benzoic acid, aromatics, and Bis (2-Ethyl) hexyl phthalate	UV, 100°C for 48h	Falah et al., 2020
*Chlamydomonas reinhardtii*	Green algae	PET film	TPA	30°C for 4weeks	Kim et al., 2020a

### Factors Affecting Microbial Degradation of PLA-PET Waste

Environmental variables, such as pH, humidity, temperature, salinity, quantity of oxygen, sunshine, water, stress, and culture conditions, have a meaningful effect on the microbial population and enzyme activity (Oliveira et al., 2020). Owing to their metabolic machinery, as well as their ability to adapt to hostile settings, microorganisms can destroy a wide range of organic contaminants. Consequently, microbes play an important role in site rehabilitation. However, their efficiency is conditional on a range of factors, including the chemical composition and quantity of pollutants, their presence in microorganisms, and the physical and chemical features of the environment. These are microbiological and genetic factors or environmental influences (Gupta et al., 2017).

When fungi grow at the lowest pH possible, the most CO_2_ is produced, and the strongest lignolytic activity occurs (Kumar and Chandra, 2020). The biodegradability of polyester is dominated by chemical factors and the physical characteristics of polyester. A significant aspect related to plastic biodegradation is the molecular weight. A low molecular weight is beneficial for biodegradation. The increases in the molecular weight of poly (e-caprolactone) diols resulted in an increase in the rate of EH of poly (e-caprolactone) diol by *Rhizopus delemar lipase* (Valderrama et al., 2019). The melting point of the polymers strongly impacts polymer enzymatic degradability. Polyester is usually more durable and less biodegradable when it has a higher melting point. Polymer degradation was decreased with the help of improvements in crystallinity and flexibility (Siracusa, 2019). Additives, stabilizers, and antioxidants used in the manufacture of polymers can delay degradation and may be toxic to microorganisms (Allen and Edge, 2021). Apart from structural considerations (linearity and branching in polymers, bond types, such as carbon–carbon, amide, and ester), the physical form of the polymer (powder, films, pellets, and fibers) may also affect a polymer’s biodegradability. Ultimately, the degradation rate is determined by the mechanism of degradation and the process acceleration (Vohlídal, 2021).

Different plastic polymers are electromagnetic radiation sensitive owing to their ability to absorb intense solar radiation (Hegazy et al., 2009). Chemical reagents included in polymeric structures can alter functional groups along with hydrophilicity/hydrophobicity (additives). It has been demonstrated that hydrogen peroxide, hydrochloric acid, sulfuric acid, and nitric acid all oxidize polymer surfaces (Moharir and Kumar, 2019). The observed increase in mineralization rate and carbon-fixing during PE degradation was ascribed to the use of chemicals that enhanced polymer oxidation (Jakubowicz, 2003).

### Potential Biogas Production Using PLA-PET Plastic Waste

Food waste is used to produce biogas, which is made up of enormous amounts of methane (Nanda and Berruti; Luo and Wong, 2019). Nevertheless, anaerobic digestion (AD) digesters are not typically used for the breakdown of plastic (Sánchez, 2020). In anaerobic conditions only, the methanogenic stage of the AD process is used for simple substrates (Treu et al., 2019; Yan et al., 2019). In addition, typical AD systems often keep the working pH at 6.8–8.0, which is not favorable for the breakdown of plastic when contrasted with a pH range with a lower value (e.g., pH ~4–6; Pathak and Navneet, 2017; Srisowmeya et al., 2020). A field emission-scanning electron microscope was used to analyze the time taken for disposable plastics (e.g., PP, PS, and HDPE) to degrade in a typical AD process, which showed that the procedure had limited success in degrading plastics (Lim et al., 2018). More recently (Zhang et al., 2018, 2019), following a 65-day anaerobic breakdown of plastic, it was revealed that the percentage of solid material in LDPE and PP was lowered by 1.5 and 2.0%, respectively. Regardless of how plastic waste is biodegraded, with some microorganisms emerging as a study subject, there is still a long way to go before petroleum-based plastics can be considered biodegradable (Baskaran and Rajamanickam, 2019; Gunawan et al., 2020; Li et al., 2020). A major research gap that has been identified is a microorganism capable of digesting plastic is as a model organism for further detailed research. Researchers should focus on finding potential bacteria that degrade plastics so that the biodegradation of plastics can be made highly efficient (Sánchez, 2020; Carmen, 2021; Matjašič et al., 2021).

The physical and chemical properties of the polymers are generally assessed through the direct observation of the anaerobic degradation process (Day et al., 1994). A number of the evaluated properties, including mass loss, mechanical property reduction, altered thermal behavior through scanning electron microscopy, infrared spectroscopy, gel permeation chromatography, and exclusion chromatography, all revealed microbial invasion (Rivard et al., 1995; Massardier-Nageotte et al., 2006; Yagi et al., 2009, 2012, 2014; Hermanová et al., 2015; Selke et al., 2015; Lee et al., 2016). These methods provide evidence of breakdown, but they cannot ascertain the degree of degradation or tell whether it was caused by microbes or other activities.

**Figure 2:**
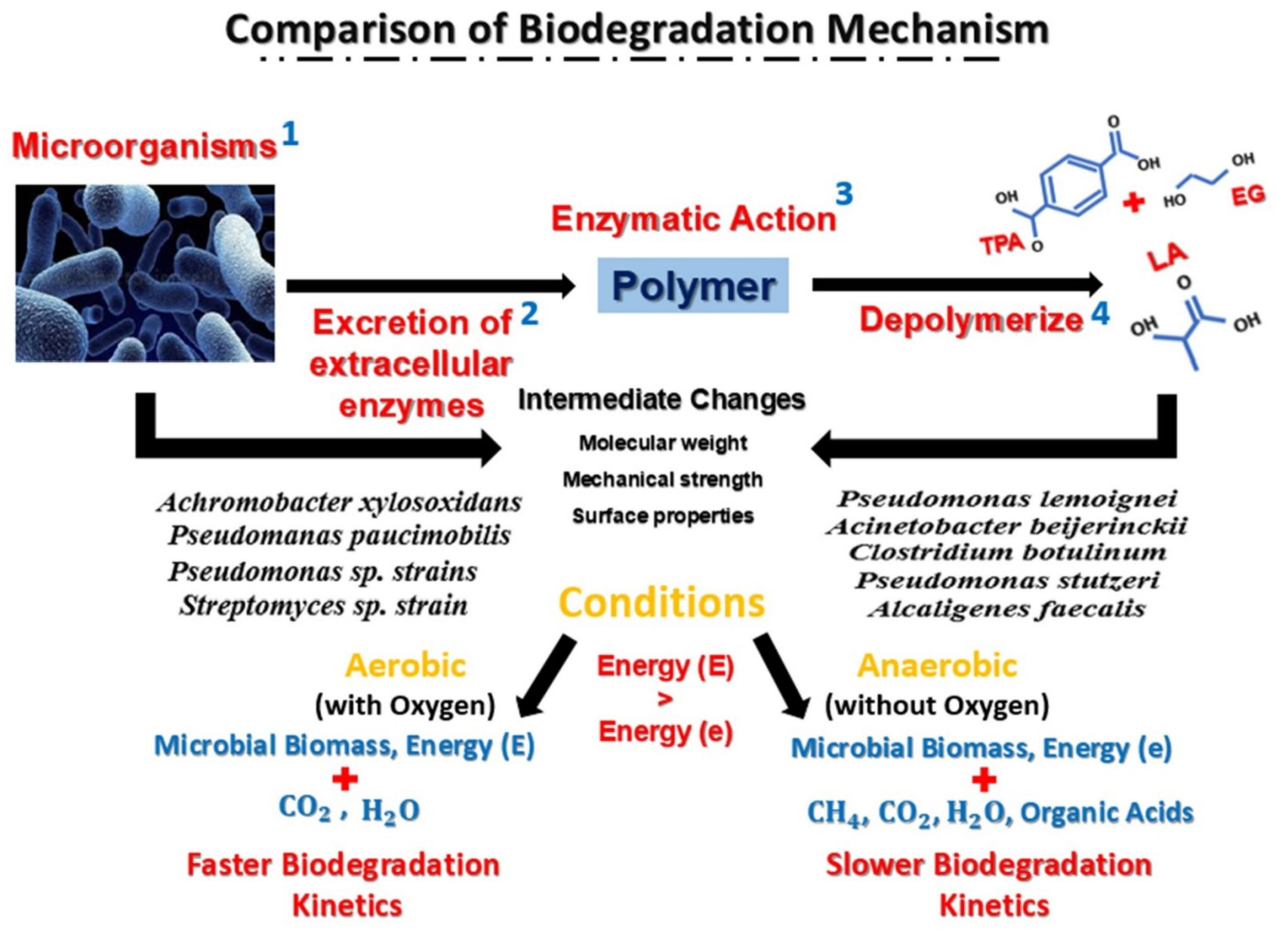
Biodegradation processes of PLA-PET plastic waste under different conditions. Biodegradation occurs under two main conditions: aerobic and anaerobic. For aerobic degradation, oxygen is necessary; in contrast, for anaerobic degradation, oxygen is needed at the start of the process, but not needed after a while. Both conditions use different enzymes that affect the molecular weight, mechanical strength, and surface properties. In one comparison, aerobic degradation occurs much faster than anaerobic degradation (Glaser, 2019). In anaerobic conditions, less energy is released compared with in aerobic conditions. The end products of the degradation are microbial biomass, CO_2_, H_2_O, and energy in aerobic digestion and extra products, namely CH_4_ and organic acids in anaerobic digestion.

Anaerobic degradation is a type of nonoxygenated biological deterioration. It uses organic matter as an electron donor or acceptor, whereas anaerobic respiration uses CO_2_, SO_4_^−2^, and NO_3_^−^. Hydration, acidification, acetogenesis, and methanogenesis are the four steps. Fermentation creates two-thirds of the CH_4_, whereas respiration produces one-third. Acidity, temperature, redox potential, and hydrogen concentration all affect anaerobic biodegradation efficiency. It also depends on the microbe concentration, nutritional levels, and substrate qualities (Mohee et al., 2008). The waste in landfills is diverse, complicating biodegradation. Anaerobic decomposition has five stages. The initial hydrolysis reduces pH, increases CO_2_ demand, and concentrates volatile fatty acids, ammonia, and sulfates. Step 2 is acidogenesis, which generates H_2_. Acetate, hydrogen, and CO_2_ are produced in Step 3. Step 4 is methanogenesis, which produces biogas. In Step 5, stabilization reduces biogas generation and increases the N_2_ concentration. Abiotic and biotic factors influence biodegradation. However, the treatment and recirculating of leachate has been demonstrated to increase biodegradation rates, enhancing the role of landfill as a bioreactor (Cossu et al., 2018). The differences between the biodegradation processes of PLA-PET waste under aerobic and anaerobic conditions are shown in Figure 2.

The breakdown of plastics results from various causes that may be physical, chemical, or biological. The mechanism is material- and condition-dependent. Exposure to UV light, temperature, mechanical stress, oxidative, hydrolytic, and biodegradative conditions causes damage. Plastics break down anaerobically in enclosed vessels or spaces; therefore, oxygen and ozone oxidation are irrelevant. For polyolefins and PS, free radicals are thought to cause consecutive cleavages along the polymeric chain. Free radicals are created through a UV-stimulated radiation process for polyethylene (Singh and Sharma, 2008).

Temperature and pH control the rate of degradation. The proposed biodegradation processes for polymers, such as PHBV, include colonization, hydrolysis, the erosion of exposed sections, saturation, and fragmentation. Microbes attack amorphous starch zones on polyethylene–starch copolymers, allowing entrance into interior holes. Therefore, the degradation of the polyethylene matrix occurs after starch consumption (Singh and Sharma, 2008). Depending on the test settings, plastics degrade at varying rates. Compared with other PHAs, this material increased biogas generation by 62%. In contrast, PHB decomposed 90% in 14days at 55°C, which was equivalent to cellulose. However, only 23% of anaerobic sludge degradation was achieved in 10weeks (Yagi et al., 2014). When eggshell was utilized to make a biocomposite, the degradation of PCL rose from 80% over 50days to negligible at 55°C. PBS was also degraded at 25.8 and 32.5% under thermophilic circumstances, but did not mineralize when tested at 55°C (Petit et al., 2015).

Polylactic acid biodegradation has been reported as anything from total mineralization to complete biodegradation, with complete biodegradation taking place in under 100days (Selke et al., 2015). The L-lactic acid treatment of PET waste resulted in a random distribution of co-monomeric units (Hermanová et al., 2015). Hydrolytic and/or biodegradable co-polyesters were created by combining aromatic and aliphatic units. Sample A generated a theoretical yield of 69% methane-rich biogas, whereas sample D generated 34%. The starting content and chain length of aromatic sequences reduced the anaerobic biodegradation rate. Notably, insoluble degradation intermediates were expected alongside soluble intermediates, depending on how long it takes to hydrolyze aromatic trimers and oligomers to dimers and monomers. Aromatic oligomers and monomers degrade in sludge.

The biodegradable starch-based polymers Mater Bi and Ecostarplus behaved differently when biodegraded anaerobically (Day et al., 1994). In the Mater Bi samples, it appeared that the starch was readily attacked by bacteria, leading to significant CH_4_ evolution and weight loss. However, the Ecostarplus polymer demonstrated minimal CH_4_ evolution and weight reduction.

Many biocomposites include starch because of its inexpensive cost, high permeability, and natural biodegradability by microorganisms. Biodegradation occurs solely for starch in polyolefin composites, indicating that these composites were not fundamentally biodegradable. The starch in these composites only partially biodegrades owing to the presence of a starch matrix. Biodegradation rates for composites with a biodegradable matrix ranged from 10.2 to 53% (Yagi et al., 2014). During polymer degradation, new products such as CO_2_, H_2_O, or CH_4_ are formed, resulting in the formation of process end products (Lucas et al., 2008). Aerobic degradation necessitates the presence of oxygen as the final electron acceptor. During the degradation of plastics, aerobic conditions lead to the production of CO_2_ and H_2_O, as well as cellular biomass from microorganisms. When polymer biodegradation occurs in sulfidogenic conditions, CO_2_ and H_2_O are produced as byproducts. Anaerobic polymer degradation yields organic acids, H_2_O, CO_2_, and methane as byproducts. Anaerobic decomposition is less efficient for energy production than aerobic degradation, involving CO_2_ and SO_4_^−2^, because of the absence of O_2_, which serves as an electron acceptor (Gottschalk, 1986). Anaerobic degradation occurs through a different method, i.e., the burial of the polymer sets off a complex chain of chemical and biological reactions. Aerobic bacteria deplete oxygen in the buried materials. The following oxygen-depleted conditions initiate anaerobic biodegradation. In most cases, 3-m–thick layers prevent oxygen replenishment. It may begin with alternative electron acceptors, such as nitrate, sulfate, or methane. Any further addition of oxygen will stop the anaerobic degradation process (Glaser, 2019).

## Environmental Waste: Biodegradable Plastic Production

Among all the environmental pollutants and contamination-causing agents, the accumulation of single-use plastic made from nonbiodegradable materials in the marine and terrestrial environments is very harmful and has toxic effects on human and marine health. Biodegradable plastic and bioplastic, which includes plastics composed of biodegradable polymers, as well as effective biobased recycling strategies offer the only realistic alternative that can achieve a safe and sustainable environment for humans and animals (Emadian et al., 2017; Abdelmoez et al., 2021). In an effort to reduce the massive plastic problem, the production and use of biodegradable plastic is more important role than other existing strategies. Many responsible countries, including the United States and China, have planned to increase the production facilities for biodegradable plastics and reduce the production of traditional, single-use plastics (Ma et al., 2020; Shen et al., 2020). Biodegradable plastics are mainly made from renewable resources, including biobased and fossil-fuel sources, such as food waste and agricultural residues (Havstad, 2020). Biodegradable plastics are the only realistic solution to the problem of plastic pollution in the environment.

### Food Waste

Food waste is one of the main spreading waste worldwide and it is considered a cheap carbon source for the production of PHAs. High-quality poly-L-lactate (PLLA) biodegradable plastic was produced from food wastes through a combination of chemical processes and fermentation. A novel system composed of D, L-lactic acid removal by *Propionibacterium* from minced food waste, which was followed by the purification, esterification, and polymerization of L-lactic acid (Sakai et al., 2003). This process produced high quantities of PLLA with high activity, but used relatively little energy and had limited emissions. Furthermore, there was no difference in the physical properties of PLLA, produced by this method and PLLA produced commercial from L-lactic acid. Researchers have put significant effort into the production of PHA from food waste as the production of PHA from food waste is the best alternative to waste disposal.

Palm oil waste has been used as a carbon source for the synthesis of mcl-polyhydroxyalkanoates (mcl-PHAs) from the *Pseudomonas* sp. Gl01 strain (Fukui and Doi, 1998). The characterization showed that this polymer consisted of C6 to C16 monomers. Another study examined genetic modifications of *E. coli* that may enhance the synthesis of PHA from cheese whey and reduce the production of organic acid. P(3HB) homopolymer and P(3HB-*co*-5mol% 3HHx) copolymer were effectively synthesized by providing soybean oil as a carbon source to *Cupriavidus necator* H16 and its recombinant strain (Murugan et al., 2016). The malt waste from a beer brewery plant, soya waste, and sucrose were also investigated substrates for the production of PHAs; malt waste resulted in the highest production of polymers (Yu et al., 1999).

### Agricultural Residues

Compared with traditional plastic, the production of biodegradable plastic consumes less energy and reduces waste, CO_2_, and greenhouse gas emissions. Biodegradable plastics produced from agricultural residue can enhance soil fertility. In the agricultural sector, biodegradable mulch films offer an environmentally friendly alternative to traditional plastic mulch film. Problems associated with traditional plastic mulch film, such as reduced fertilizer effectiveness, poor soil fertility, and malnutrition, can be prevented by using biodegradable mulch film. The production of biodegradable plastic from agricultural residues for the mulch film is an interesting solution to related problems. The plastic products used in our daily life can be produced from polymers that can rapidly degrade, reducing the pressure of landfill sites. Biodegradable polymers produced from renewable resources are likely to prove irreplaceable in high-value markets, such as medical sutures, drug-delivery materials, and orthopedic needles (Shen et al., 2009, 2020).

The production of PHA using cane molasses and the waste from ethanol production as a substrate has been reported from different parts of plants (Verlinden et al., 2007; Khiyami et al., 2011). PHB was produced from wheat straw and bagasse hydrolysate, which are renewable sources of agriculture residue (Yu and Chen, 2008; Galia, 2010). Xie et al. (2020) also studied the preparation of biopolymers film containing potato peel powder, curcumin, and bacterial cellulose. Their results showed that the incorporation of bacterial cellulose in the PP matrix increased the mechanical properties of the biopolymer films, reduced water vapor and oxygen permeability, and reduced light transmission.

### Accumulating Bacteria

Scientists in this area of research prefer the use of microbes for the production of biodegradable plastic owing to their easy handling, maintenance, purification, and susceptibility to genetic modification for the desired properties (Mostafa et al., 2020; Narayanan et al., 2021). Polyhydroxybutyrates are macromolecules synthesized by bacteria. These are inclusion bodies accumulate when bacteria are subjected to stress. Polyhydroxybutyrates have similar properties to synthetic thermoplastics and good mechanical, physical, and immunological properties that make them an excellent biopolymer (Verlinden et al., 2007; Galia, 2010; Khiyami et al., 2011). To achieve industrial production, different microorganisms are being studied for their ability to produce different qualities and quantities of polyhydroxybutyrates with different decomposition rates (Natarajan, 2011; Juengert et al., 2018). Bacteria are most often used for their fabrication as they are able to produce the greatest biomass in the shortest time, and have simple downstream processes and more possibilities for gene modification compared with fungi, yeasts, and algae (Kumarasamy et al., 2020; Soman et al., 2020a,b). *Azoto-bacter vinelandii*, *E. coli*, *Pseudomonas oleovorans*, *Halomonas campaniensis*, *Alteromonas lipolytica*, *Aeromonas* sp., *Bacillus megaterium*, *Methylobacteria* sp., *Herbaspirillum seropedicae*, and *Alcaligenes latus* are the most commonly used of the 300+ bacterial species that can accumulate polyhydroxybutyrates and polyhydroxyalkanoates (Rendón-Villalobos et al., 2016; Shi et al., 2017; Tufail et al., 2017; Gabr, 2018). Potential PHB-producing bacterial strains were isolated and the production of PHB was evaluated using agricultural residues as the carbon source (Getachew and Woldesenbet, 2016). The optimum conditions for PHB production were pH 7, 37°C, and an incubation period of 48h with shaking at 120rpm.

## Pyrolysis: Waste Into Energy

Oil can be described as a dark blood that flows through the veins of the global elite. As a major power source, it has brought prosperity to the developing world. However, the continued resources of oil are uncertain and can be expected to fluctuate dramatically owing to restrictions on supply. Power is important to all areas of society. The major improvements and developments in society over the past 200years have been driven by the use of nonrenewable energy resources. The maximum usage of petroleum products will soon be reached. Therefore, sound strategies for determined new sources are essential, especially for unrefined oil. It is important to consider several sources potential sources of oil; however, to obtain a realistic viewpoint, the factors that affect the production of hydrocarbons must be considered. The use of plastic waste and EH may play an important role in the production of bio-oil. In addition, changing the structure or the surface functionality of plastic using enzyme hydrolysis may improve the quality and yield of pyrolytic bio-oil.

### Thermal Pyrolysis of PLA-PET

**Figure 3:**
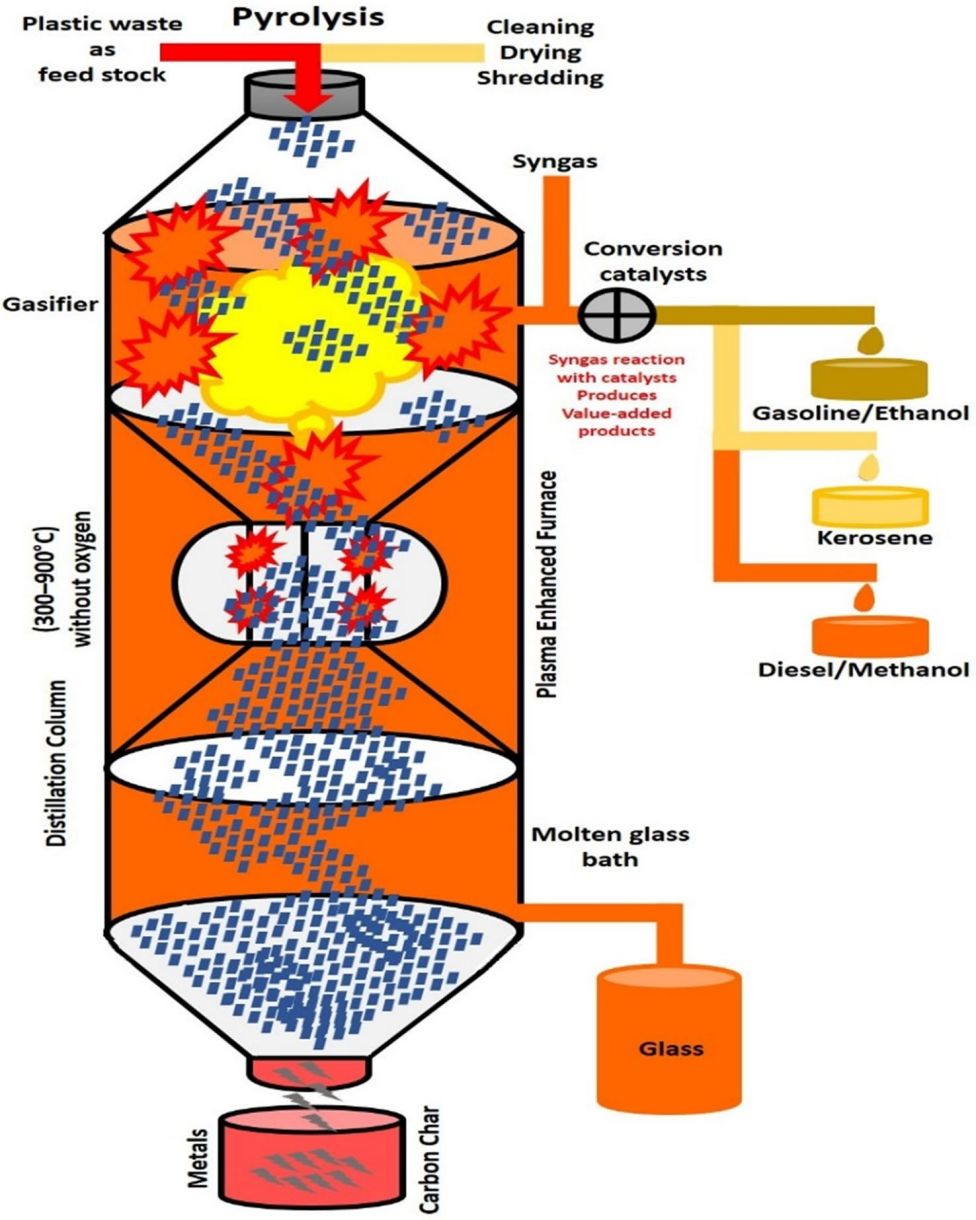
Pyrolytic process for the conversion of plastic waste into energy. Plastic to oil or other fuel (PTF) conversion technology is discussed here, as well as the possible reductions in CO_2_ emissions and other environmental advantages that might result if the Resources Recovery Authorities implemented these technologies. Plastics can be converted to oil or other fuels *via* pyrolysis. Before pyrolysis, plastic waste is shredded and cleaned for pretreatment, followed by the conversion of plastic waste to gas. The gas is converted to a liquid *via* distillation, and the acids formed during the breakdown of certain scrap plastics will be removed to prevent any corrosion of the PTF systems and engines. The final steps are the separating, refining, and blending of the fuel.

Polyethene terephthalate is an essential substance that is abundant in a variety of areas, such as food, electrical cables, and scientific equipment. Bioaccumulation and poisonous gasoline when igniting are common problems (Zimmermann, 2020). Therefore, as a necessity, the problem of plastic waste needs to be solved to prevent climate change and to improve human health. The typical recycling processes for plastic waste are shown in Figure 3. There are exclusive disposal strategies for PET waste, although all modern techniques have regulations. For example, incineration is a regular disposal method for solid urban waste (MSW; Kim et al., 2010). Although it may not be difficult to handle, and that it prevents an opportunity to reduce plastic waste, air pollutants such as dioxins, furans, mercury, and polychlorinated biphenyls are released (Diaz-Silvarrey et al., 2018). It could also result in the destruction of potential material that could have been disposed of as PET waste (Verma et al., 2016). In addition, constructs that live after PETs have been burned pollute the soil by leaching out metals such as lead and cadmium (Raheem et al., 2019). Reuse is also an option, and is considered as it is very environmentally friendly and prevents the destruction of items (Jamdar et al., 2017). In addition, sorting plastic before reuse requires extra labor (Gurgul et al., 2018). Therefore, a more feasible and reliable approach to PET waste disposal is required.

Another treatment method is pyrolysis, i.e., thermal treatment without oxygen (Kim et al., 2020c). This method has been used for various waste materials, including plastic (Kwon et al., 2019). The advantages are that it can reduce the amount of waste and transfer the estimated substances introduced by means of combined heat and power (Kim et al., 2020c). Valuable objects such as pyrolysis oil and gasoline have too great a thermal power, and this can be converted into a pyrolysis plant to satisfy the power requirements (Bridgwater and Peacocke, 2000). Heat treatment is considered because it is preferred not to require complex skills or extreme work cycles; moreover, it could not use plastic in any way like traditional techniques (Adrados et al., 2012). Although pyrolysis has several advantages over other methods of plastic waste recycling, its drawback is the generation of hazardous substances, such as polycyclic compounds and biphenyls, which can have to serious impact on ecological systems and human health if not properly managed (Kim et al., 2020d). For example, polycyclic aromatic compounds (PAHs) can be exchanged with particulate oxygen-containing PAHs, which are sometimes present in natural smoke concentrates, through a reaction with NOx and O_3_ (Gbeddy et al., 2020). To ensure a strong, natural approach to PET waste disposal, the whole process must be considered and polycyclic fragments should be avoided.

The pyrolysis of reagents has been used to study thermal efficiency and the incorporation of alternative pyrolytic objects (Lee et al., 2020). Although most studies on the pyrolysis of reagents have used zeolites, because of the current impetus to increase the yield of bio-oil and, similarly, to increase the reaction yield (Kim et al., 2019a). In addition, owing to the coke reinforcement, zeolites are not available at excessively high temperatures (Kim et al., 2019b). Upheld’s decent metal pulses are thermally stable and show excessive movement at excessive temperatures (Kim et al., 2020b); however, from now on they can be used in pyrolysis, which is conducted at high temperatures. For example, a Pt pulse, maintained in activated carbon, has been used extensively in the pyrolysis of food waste to reduce the build-up of harmful artificial species during pyrolysis (He et al., 2012).

### Catalytic Fast Pyrolysis of PLA-PET

The biotreatment process of plastic waste is believed to enhance the performance of pyrolysis, including increased thermal conversion rate and selectivity, and a decrease in the required temperature. Many recent reports have discussed the importance of enzymatic treatment before the pyrolysis process for lignin, which is high molecular weight insoluble polymer. Unfortunately, there are no reports discussing the importance of enzymatic treatment before thermal pyrolysis of plastic waste. Enzymatic treatment of plastic waste before thermal pyrolysis can help to decrease the activation energy and pre-exponential factor of the thermal pyrolysis of plastic waste. Wang et al. (2020) used the enzymatic treatment of lignin before thermal pyrolysis, which significantly enhanced the performance and selectivity of pyrolysis at lower temperatures and decreased the activation energy.

Thermochemical measures (ignition, gasification, pyrolysis, and torrefaction) are among the best techniques to gain additional energy from starch biomass (Bridgwater, 2012). One of the more promising thermochemical methods, pyrolysis, produces a liquid powder. Rapid pyrolysis occurs between 300 and 500°C in an oxygen-free or oxygen-restricted atmosphere with high heat loads (>1,000°C/min) and a rapid vapor cooling period. Bio-oil yields in the range of 60–70% by weight cannot always be realized.

Bio-oil is a complex natural mixture that contains low-molecular-weight biomass. Normally, it is refined for different utilizations (Bridgwater et al., 2009). Lignocellulosic biomass feedstocks, such as rural deposits, are a water-based mixture of cellulose, hemicellulose, lignin, extractives, and minerals (P, K, Ca, Mg, and Na).

Many of these minerals help to catalyze secondary responses that alter the yields of liquid, solid, and gaseous products during fast pyrolysis (Akhtar and Amin, 2012). Excellent organic oil at some point in the pyrolysis depends on the desired product and the degree of oxygen-containing compounds, specific conditions were used, particularly to intensify the deoxygenation reactions at a certain stage of the pyrolysis, e.g., Zeolites and steel oxides (Zhang et al., 2009). After the oil has accumulated, various measures, such as hydrocracking, esterification, reagent degradation, etc. (Goldberg et al., 2015) were used to refine the bio-oil. In addition, the oil fit is critical to capacity and transportation so that it does not deteriorate with unpredictable additional polymer designs (Carlson et al., 2008). In one model, Atadana (2010) performed pyrolysis of the reagent with HZSM5 zeolite in biomass washed with NaOH to improve the yields of levoglucosan and caramel odor compounds. It was found that this reaction drove the formation of water at a certain stage of pyrolysis, which results in an excessive moisture content in the oil. In addition, zeolite pulses are quite expensive, which makes a cheaper alternative very attractive.

Lu et al. (2010) studied metal oxide nanoparticles in pyrolyzed wood waste (MgO, CaO, TiO_2_, Fe_2_O_3_, NiO, and ZnO). When all the solids were removed, it was discovered that the calcium oxide underwent major alterations and that the admission of anhydrous phenols, acids, and sugars had stopped. Pen tested the effect of calcium and potassium salts in the pyrolysis of biomass and a large number of carboxylic acids and furans and a lower oxygen content in the oil (Goldberg et al., 2015). Mullen et al. (2011) conducted a brief pyrolysis of sawdust in a fluidized-bed reactor using two successive Ca pulses. The two CO and CO_2_ pulses used in conjunction decreased the oxygen concentration of the oil. In addition, they found a reduced mean output with natural caustic. Wang et al. (2010) conducted investigations with two pulse types: acid zeolite MCM41 and essential CaO. The pyrolysis was performed on a TGA-FTIR for the advantageous cluster area, although it is not always a short pyrolysis because the heating load is only 90°C/min. Their results verified that the yield of corrosives decreased, along with a range of hydrocarbons. Acid production was reduced by the use of CaO, and the CaCO_3_ that was created had the potential to degrade into ketones and water. Calcium oxide has been reported by Lin et al. (2010) to induce the pyrolysis of pine sawdust in a fluidized-bed reaction on the scale of a study facility (180–1,000g/h). The percentage of biomass/pulse was modified (0–30%). The yields of formic acid, levoglucosan, phenol, acetone, and guaiacol decreased as the content of CaO was increased, and various mineral impulses were also tested (Beaumont and Schwob, 1984). Overall, reactive pyrolysis is attractive when you consider that it benefits from the warm inert temperature of the vapors after pyrolysis.

During a synergistic *in situ* pyrolysis the impulse fragments are mixed with (Lin et al., 2009) or essentially displace the fluidizing medium (Williams and Nugranad, 2000). Unfortunately, this type of association typically has problems with catalyst deactivation and requires chronic pulse regeneration and replacement. In contrast, *ex situ* reagent pyrolysis structures generally have a solid cushion after the fluidization sector of the reactor. At the same time, this type of structure has much smaller deactivation momentum, which increases domestic smoke cases and improves response to polling breaks. This shows the potential of *in situ* synergistic pyrolysis to provide higher caliber oils.

### Challenges Associated With Thermal Pyrolysis of PLA-PET

Recycling plastic waste has emerged as one method of reducing plastic pollution; however, this generally requires large labor costs and, at some stages in processing, create ignited water. If plastic recycling can support the liquids with tribological effects, such as lubricating oils, this is a convenient pathway to eliminate plastics in the environment. To understand potential tribological uses of recycled plastic, it is important to study techniques that can be used to convert waste plastic into completely petroleum-based oils. The viscosity, density, and friction of pyrolyzed waste plastic oils are examined and evaluated in comparison to commercial lubricants to determine their function and lubrication effects. Segregation strategies, catalytic isomerization dewaxing, and the Fischer-Tropsch method for recycling plastic waste have also been examined to obtain insight into techniques for converting pyrolyzed plastic waste into lubricants (Sikdar et al., 2020).

The separation of other plastics from PLA and PET can be difficult can be difficult; naturally, this impacts the ability to recycle these materials. The purpose of this test is to examine the feasibility of recycling mixed PLA and PET waste *via* a copyrolysis method. Samples containing significant levels of pre-processed PLA and PET waste were subjected to the relevant studies using thermogravimetric (TG) and reaction kinetic analyses. Finally, pyrolytic studies based solely on the determined TG reaction conditions were completed to investigate the yields of the pyrolytic reactions. The results indicated that the HHV of PLA and domestic animal was approximately 18.26 and 22.85MJ/kg, respectively, and that of the combinations fell between those values. Each sample has a combustible fraction of more than 96% and a maximum decomposition temperature between 618 and 736K. Additional PET ratios were determined to lead to higher activation energies and pre-exponential elements. In addition, the PLA ratios were correlated with the mass yield of carbonated goods, whereas the PET ratios were correlated with the yields of resistant and condensing products. To avoid potential issues with strength and performance, the copyrolysis of PLA and PET can also mitigate the need to decompose PLA and PET, which can contribute to a reduction in plastic waste (Tai, 2015).

The linear economic system for plastic packaging, which currently results in the emission of too much CO_2_ and the release of plastic into the environment, needs to be reformed into a more spherical and ecologically focused model that is resource- and environmentally friendly throughout the plastics value chain, from product develop, to recycling, and with the abandonment of existing alternatives. This review highlights areas in which those who are inexperienced in chemistry can make contributions. Replacing plastics derived from fossil fuel sources with fully renewable biobased options can reduce greenhouse gas emissions and produce plastics that are much less complex to recycle and biodegrade at the end of their useful existence in the environment. The underlying chemical and biocatalytic production for plastic production and recycling has been reviewed and priorities for future improvements have been identified (Sheldon and Norton, 2020).

Polymers such as polyamides or nylon, PET, polycarbonate (PC), and polyurethane (PE) are known as condensation polymers because, in addition to carbon (C) atoms, they consist of certain atoms such as oxygen (O) and nitrogen (N). They can be degraded by hydrolytic cleavage as hydrophilic ester or amide bonds are present in their monomers. Biodegradation begins at the same time that the molecular weight of the polymers reaches several thousands, which is usually a low value for commercial polymers (Bonhomme et al., 2003). Another difficulty affecting the biodegradation and pyrolysis of polymers is their crystallinity. The specific physical phenomena that occur within the pyrolysis pathway depend on the material. Thermosetting polymers are insoluble and therefore their modification phase is not feasible during pyrolysis. However, thermoplastics can be softened within the pyrolysis reaction with reversible phase modifications until a minimum pyrolysis temperature is reached (Brostow and Lobland, 2016). There are no completely crystalline polymers, but there are completely amorphous polymers. A crystalline material under a given voltage has a single melting temperature, but no variety; similarly, the glass transition occurs over a variety of temperatures, but not at a single temperature.

The biodegradation of polymers is based on the chemical and physical properties of the polymer. The crystallinity and molecular weight of the polymer are key factors that help biodegradation. Increasing the molecular weight of a polymer reduces its degradability (Tokiwa et al., 2009). For example, polymeric polycaprolactone (PCL) with a molecular weight of >4,000 is slowly degraded by a hydrolysis pathway using a *Rhizopus delemar* lipase, unlike PCL that has a lower molecular weight. Various enzymes from microorganisms are a useful resource for the biodegradation of polymers through oxidation and hydrolysis processes. Crystallinity also affects the rate of biodegradation in biopolymers. In the absence of additives, many commercial polymeric substances may encounter obstacles to their use. However, it was found that additives such as phthalates and bisphenol can damage human health through their impact on natural reactions (Meeker et al., 2009).

## Conclusion and Future Perspective

The enormous problems arising from the accumulation of different plastic wastes has attracted the attention of scientists worldwide. The correct use of this waste may be a “hidden treasure,” especially for the conversion and production of various value-added products. A variety of plastic-degrading enzymes have been recently discovered, widening the potential applications for mixed-plastic waste biodegradations. Moreover, there is a very urgent need for safe and nontoxic alternatives to fossil fuel-based plastics, such as biodegradable plastics like polyhydroxyalkanoates, which can be more easily degraded by microorganisms. Diethyl-2,5-furandicarboxylate and diethyl isophthalate are also promising alternatives to fossil fuel-based plastics and can be easily produced by enzymatic catalysis. Biodegradable plastics can be produced from food waste, agricultural residues, and polyhydroxybutyrate-accumulating bacteria. The thermal pyrolysis of mixed-plastic waste is also a very important method that may overcome the depletion of natural crude oil. A possible hybrid integration technique to incorporate thermostable plastic degradation enzymes in the conversion into fuel oils requires further investigations. The discovery of highly thermostable enzymes is essential for the development of mixed-plastic waste thermal pyrolysis.

## Author Contributions

MT, NS, YJ, MM, HS, MB, and JX wrote the manuscript. MT drew all the figures. NS and JX designed the paper concept and revised the manuscript. All authors contributed to the article and approved the submitted version.

## Funding

This article acknowledges the funding support provided by the National Natural Science Foundation of China (grant numbers 31961133017, 31961133018, and 31961133019). These grants are part of the “MIXed plastics biodegradation and UPcycling using microbial communities” MIX-UP research project, which is a joint NSFC and EU H2020 collaboration. In Europe, MIX-UP has received funding from the European Union’s Horizon 2020 research and innovation program under grant agreement no. 870294.

## Conflict of Interest

The authors declare that the research was conducted in the absence of any commercial or financial relationships that could be con.strued as a potential conflict of interest.

## Publisher’s Note

All claims expressed in this article are solely those of the authors and do not necessarily represent those of their affiliated organizations, or those of the publisher, the editors and the reviewers. Any product that may be evaluated in this article, or claim that may be made by its manufacturer, is not guaranteed or endorsed by the publisher.
